# Tumorigenic and tumoricidal properties of exosomes in cancers; a forward look

**DOI:** 10.1186/s12964-024-01510-3

**Published:** 2024-02-15

**Authors:** Zahra Abbasi-Malati, Seyed Ghader Azizi, Soheil Zamen Milani, Zeinab Aliyari Serej, Narges Mardi, Zahra Amiri, Zohreh Sanaat, Reza Rahbarghazi

**Affiliations:** 1 Department of Applied Cell Sciences, Faculty of Advanced Medical Sciences, Tabriz University of Medical Sciences, Tabriz, Iran; 2 Clinical Immunology Research Center, Zahedan University of Medical Sciences, Zahedan, Iran; 3 Student Research Committee, Tabriz University of Medical Sciences, Tabriz, Iran; 4 Biotechnology Research Center, Tabriz University of Medical Sciences, Tabriz, Iran; 5 Department of Tissue Engineering, Faculty of Advanced Medical Sciences, Tabriz University of Medical Sciences, Tabriz, Iran; 6 Hematology and Oncology Research Center, Tabriz University of Medical Sciences, Tabriz, Iran; 7 Stem Cell Research Center, Tabriz University of Medical Sciences, Tabriz, Iran

**Keywords:** Exosomes, Oncogenic properties, Resistance mechanisms, Tumoricidal effects, Stem cells

## Abstract

In recent decades, emerging data have highlighted the critical role of extracellular vesicles (EVs), especially (exosomes) Exos, in the progression and development of several cancer types. These nano-sized vesicles are released by different cell lineages within the cancer niche and maintain a suitable platform for the interchange of various signaling molecules in a paracrine manner. Based on several studies, Exos can transfer oncogenic factors to other cells, and alter the activity of immune cells, and tumor microenvironment, leading to the expansion of tumor cells and metastasis to the remote sites. It has been indicated that the cell-to-cell crosstalk is so complicated and a wide array of factors are involved in this process. How and by which mechanisms Exos can regulate the behavior of tumor cells and non-cancer cells is at the center of debate. Here, we scrutinize the molecular mechanisms involved in the oncogenic behavior of Exos released by different cell lineages of tumor parenchyma. Besides, tumoricidal properties of Exos from various stem cell (SC) types are discussed in detail.

## Introduction

Cancer diseases have been debilitating conditions in human medicine in the last decades with high-rate morbidity and mortality [1]. In clinical settings, surgical approaches, chemotherapy, radiation, and neo-adjuvant therapies are still effective strategies for early cancer treatment [2]. Despite recent advances in cancer theranostics, tumor heterogeneity increases the probability of drug resistance, leading to treatment failure and cancer recurrence [3]. During the last decades, the advent and development of stem cell-related technologies have led to prominent progress in the treatment and alleviation of several pathological conditions [4]. Different stem cell types, including embryonic stem cells (ESCs), induced pluripotent stem cells (iPSCs), and adult stem cells exhibit differentiation capacity to several lineages, making them as valid cell source for restoration of injured cells [5]. Among different stem cell types, adult mesenchymal stem cells (MSCs) have been extensively applied in various diseases with eminent regenerative outcomes. However, data confirmed that small fractions of transplanted MSCs are alive after direct introduction into the injured sites or a very low cell population can be recruited into the targeted sites after systemic administration [6, 7]. In light of these outcomes, it is believed that MSC therapeutic properties are mainly associated with the paracrine capacity and release of diverse signaling molecules such as cytokines, interleukins (ILs), growth factors, etc. via extracellular vesicles (EVs) into the extracellular matrix and biofluids [8]. Besides these facts, the direct injection of stem cells is associated with a short lifespan and survival rate, off-target delivery, infusion toxicity, activation of allogeneic immune cells, and various malignancies [5]. Meanwhile, the isolation and expansion of stem cells are laborious and expensive and the possibility of genetic and epigenetic instabilities, and loss of stemness are the main challenges in the clinical setting [9]. These features increase the application of stem cell secretome as an alternate to whole-cell-based therapies in clinics with at least biosafety concerns [10].

**Fig. 1:**
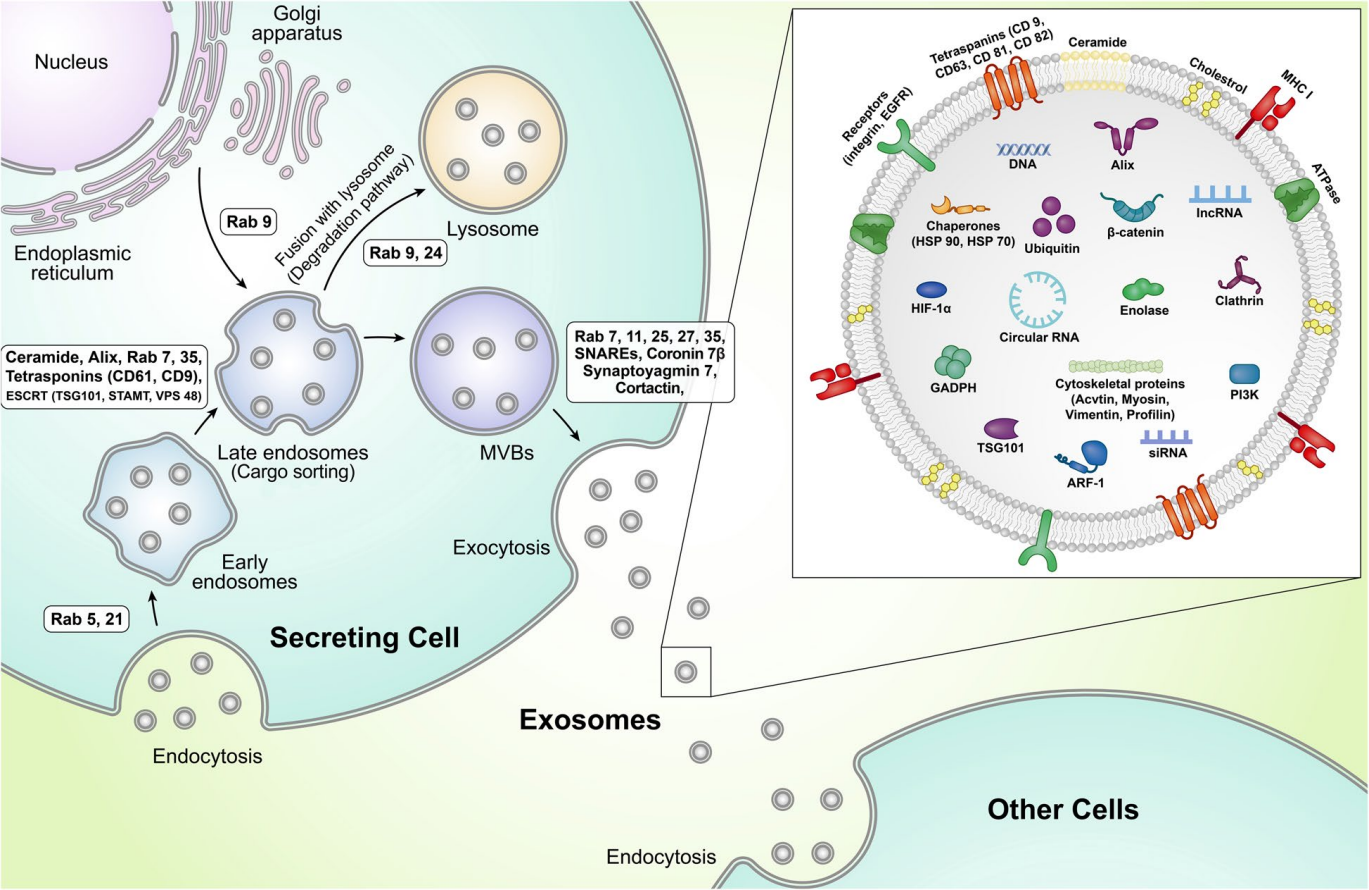
Biogenesis of exosomes (Exos). The endosomal system is actively involved in the generation of Exos. The internalized Exos are sorted into early endosomes. In the next steps, these endosomes mature into late endosomes and MVBs where new nano-sized vesicles, ILVs, are generated via the invagination of the endosomal membrane. These particles are named Exos upon their release into the ECM. MVBs can direct lysosomal degradation to directly fuse with the cell membrane to release their contents into the ECM. Abbreviations: MHC1: major histocompatibility complex 1, CD: Cluster of differentiation, EGFR: Epidermal Growth Factor Receptor, HSP: Heat shock proteins, HIF-1α: Hypoxia-inducible Factor 1α, GAPDH: Glyceraldehyde-3-phosphate dehydrogenase, TSG101: Tumor susceptibility gene 101, ARF-1: ADP-ribosylation factor 1, PI3K: Phosphoinositide 3-kinases, Rab: Ras-associated binding, SNARE: soluble N- ethylmaleimide- sensitive fusion attachment protein receptor, MVB: Multivesicular Body, ESCRT: Endosomal sorting complexes required for transport, STAM: Signaling transducing adaptor molecule, VPS4: Vacuolar protein sorting 4, ALIX: ALG-2-interacting protein X

Exos with lipid bilayer membrane and nano-sized dimensions (30–150 nm) have the potential to carry several signaling molecules between the cells in a paracrine manner [11, 12]. Exos can easily be distributed in several biofluids such as blood, urine, saliva, and other biofluids, reflecting the metabolic status of parent cells [13]. A long with these comments, the origin and metabolic status of parent cells can pre-determine exosomal cargo under different conditions such as cancers [14, 15]. Emerging data have revealed the critical role of Exos in the dynamic growth of cancer cells. These magic bullets can orchestrate cell-to-cell crosstalk within the tumor microenvironment (TME) to regulate tumor mass expansion and cancer cell survival. Such functions can control the development of cancer stem cells (CSCs), TME remodeling, angiogenesis, and invasion of remote sites [16]. Unlike oncogenic properties, Exos can also exert tumoricidal effects on cancer cell lineages [17, 18]. These features make the Exos suitable alternates for tumoricidal therapies. Using smart loading techniques and surface modifications, specific therapeutics can be loaded onto the Exos with appropriate on-target effects [19]. To be specific, Exo-drug delivery can reduce side effects and off-target toxicity following direct administration of chemotherapeutics [20]. In this regard, engineered Exos can intelligently deliver the therapeutic cargo to the targeted sites and diminish the possibility of drug resistance issues (Table 1) [21]. The ability to cross several natural barriers such as blood–brain-barrier etc. makes the Exos superior to synthetic nanoparticles in terms of drug delivery purposes [22]. As above-mentioned, Exos can harbor several signaling molecules that are identical to the parent cells. The Exo molecular signature can be used as a platform for early-stage detection of anaplastic changes, progression, and follow-up of the therapeutic protocols (Fig. 1) [23]. For example, CSC-derived Exos exert pro-oncogenic effects on the non-CSC lineages and normal cells. Monitoring these Exos and their contents can give us invaluable data about the dynamic growth of tumor cells within the tumor mass [24]. Here, the tumorigenic and tumoricidal properties of Exos will be discussed in different cancer types focusing on the possible molecular mechanisms. Recent advances in the application of stem cells Exos in cancer therapy were also highlighted as cell-free therapeutic approaches in cancer therapy.

**Table 1:** The exosomal cargo mediating drug resistance in different types of cancers

	**Biomarkers**	**Cancer type**	**Target molecule (s)**	**Drug**	**Reference**
**miRs**	miR-21	esophageal squamous cell carcinoma	STAT3	cisplatin	[25]
	miR-378-3p & miR378d	Breast cancer	EZH2/STAT3	doxorubicin & paclitaxel	[26]
	miR-205	Breast cancer	E2F1	tamoxifen	[27]
	miR-4443	non-small cell lung carcinoma	FSP1	cisplatin	[28]
	miR-3173-5p	Pancreatic ductal adenocarcinoma	ACSL4	gemcitabine	[29]
	miR-21-5p	Breast cancer	S100A6	doxorubicin	[30]
	miR- 301b-3p	Gastric cancer	TXNIP	cisplatin/ vincristine	[31]
**lncRNAs**	SNHG7	Lung adenocarcinoma	PI3K/AKT	docetaxel	[32]
	CCAL	colorectal cancer	β-catenin	oxaliplatin	[33]
	H19	non-small cell lung cancer	miR-615-3p/ATG7	erlotinib	[34]
	Linc00969	Breast cancer	-	trastuzumab	[35]
	UCA1	non-small cell lung cancer	miR-143/FOSL2	gefitinib	[36]
	FOXD3-AS1	Lung cancer	PI3K/Akt	5-fluorouracil	[37]
	HOTAIR	glioblastoma	miR-519a-3p/RRM1	temozolomide	[38]
	PICSAR	cutaneous squamous cell carcinoma	miR-485-5p/REV3L	cisplatin	[39]
**circRNAs**	circWDR62	glioma	miR-370-3p/MGMT	temozolomide	[40]
	circDLGAP4	Neuroblastoma	miR-143/HK2	doxorubicin	[41]
	hsa_circ0014235	non-small cell lung cancer	miR-520a-5p/CDK4	cisplatin	[42]
	circVMP1	non-small cell lung cancer	miR-524-5p-METTL3/SOX2	cisplatin	[43]
	circUSP7	non-small cell lung cancer	miR-934/SHP2	anti-PD1	[44]
	circSYT15	cervical cancer	miR-503-5p/RSF1	cisplatin	[45]
	circ-PVT1	gastric cancer	miR-30a-5p/YAP1	cisplatin	[46]
**Proteins**	MMP14	Pancreatic ductal adenocarcinoma	-	gemcitabine	[47]
	HSP gp96	Breast Cancer	p53	paclitaxel	[48]
	EGFR	non-small cell lung cancer	PI3K/AKT and MAPK	osimertinib	[49]
	FOSL1	colorectal cancer	ITGB4	oxaliplatin	[50]
	MIF	glioma	PI3K/AKT	temozolomide	[51]
	TPX2	non-small cell lung cancer	WNT/β-catenin	docetaxel	[52]
	CD44	Breast Cancer	-	doxorubicin	[53]

## Exo biogenesis

Exos are produced by the activity of the endosomal system via engaging several signaling molecules [54]. The phenomenon of Exo biogenesis is promoted by the engulfment of recently internalized Exos via endocytosis inside the early endosomes or fusion of trans-Golgi network vesicles with later endosomes or multivesicular bodies (MVBs) (Fig. 1) [55]. The endosomal system is promoted by the maturation of early endosomes into later endosomes and MVBs. Inside the later endosomes and especially MVBs, invagination of the vesicular membrane leads to the formation of numerous intraluminal vesicles (ILVs) [56]. This phenomenon is regulated by the participation of several proteins and factors that help to simultaneous sequestration of signaling molecules into the lumen of ILVs [57]. Molecular investigations have revealed the crucial role of endosomal sorting complex required for transport (ESCRT)-dependent and ESCRT-independent complexes in the formation of ILVs and cargo sorting [58]. The ESCRT complex is composed of four subunits, I, II, and III, with auxiliary factors including vacuolar protein sorting 4 (VPS4), vesicle trafficking 1 (VTA1), and ALG-2-interacting Protein X (ALIX). The close interaction of these factors leads to the sorting of ubiquitinated molecules into the ILVs [59, 60]. The sorting of non-ubiquitinated cargos is mediated via a non-conventional ESCRT-dependent complex which is composed of Syndecan-Syntenin-Alix-ESCRTIII [55, 61]. Besides these factors, tetraspanins (CD63, CD81, and CD9), and sphingomyelinase 2 enzymes (nSMase 2) are involved in the sorting of non-ubiquitinated molecules into the ILVs [62–64]. To be specific, tetraspanins are located in the endosomal membrane microdomains with key roles in the invagination of membrane and sorting of special proteins and intracellular factors into MVBs [64, 65]. Neutral sphingomyelinase 2 (nSMase 2)-enriched microdomains via conversion of endosomal membrane sphingomyelin to ceramide, induction of negative curvature of and formation of cone-shaped structure lead lateral separation vesicular membrane and formation of ILVs [66]. Inside the cytosol, the activity of different Ras-associated binding (Rab) GTPase types orchestrates the intracellular transport of endosomes [67]. Depending on the activation of specific Rabs, MVBs can be directed toward lysosomal degradation and release of cargo into the host cells. In alternative pathways, MVBs can be guided toward the Golgi apparatus or fuse with the cell membrane to release the content into extracellular matrix (ECM) [68]. The activation of Rab9 can contribute to endosomal trafficking to the Golgi apparatus while Rab7 increases the lysosomal degradation via inter-endosome-lysosome connection [69]. It should not be forgotten that the activation of similar Rab type in normal or cancer cells yields different outcomes in terms of MVBs destination. For instance, Rab7 activation in cancer cells enhances ILV secretion into the ECM [70]. Other GTPases such as Rab27a and Rab27b promote physical connection, tethering, and fusion of MVBs with cell membranes [71, 72]. Other Rabs such as Rab3, Rab11, and Rab35 are involved in endosomal recycling and ILV cargo secretion [73, 74]. Along with the activation of the Rabs, the soluble NSF Attachment Protein Receptor (SNARE) complex (SYX-5, YKT6, vesicle-associated membrane protein (VAMP)3/7, SNAP23) strengthens the fusion of MVBs with the plasma membrane [55] (Fig. 1). Upon the release of ILVs into ECM, these nanoparticles are hereafter Exos.

## Oncogenic and anti-oncogenic properties of Exos

### Oncogenic properties of Exos

#### Exos and TME

Some studies have indicated the transfer of different oncogenic products in the lumen of Exos and their influences on tumorigenesis via engaging several mechanisms [75]. For example, proteins related to the Ras superfamily of GTPases, and mRNAs of H-ras and K-ras, along with several oncomiRNAs were detected in prostate cancer cell Exos [76]. It is also possible that nucleus and mitochondria DNA are sorted into Exos inside the cancer cells in the levels of these elements were higher in cancer Exos than that of normal cells [77]. TME remodeling and stimulation of several anti-tumor activities such as polarization of macrophages toward M2 type are induced in the presence of miRNA-21A bearing cancer cell Exos. In lung cancer cells, this miRNA can directly target the programmed cell death protein 4 and inactivate myeloid-derived suppressor cells (MDSCs) [78]. Exos can play a certain role in TME for cell-to-cell intercommunication via a paracrine manner and regulation of tumor cell metastasis, angiogenesis, and immune cell function [16]. Of note, it should not be forgotten that the production and release of Exos from cancer cells is higher compared to normal cells [79]. Therefore, one can hypothesize that the role of paracrine interaction between the cells is more prominent compared to normal cell counterparts. As a common belief, tumor cell Exos are uptaken by neighboring tumor cells, CSCs, endothelial cells (ECs), and immune cells [80]. The existence of specific cargo inside the Exos can lead to stimulation of certain signaling pathways inside the tumor cells. For example, it was indicated that signaling cascades such as JAK/STAT3, KIT/ERK/BCL2, KIT/ERK/Akt/mTOR, KIT/PI3K/Akt/mTOR, HGF/MET/RAF1/MEK, HGF/MET/PI3K/Akt/mTOR and PDCD1/mTOR are the targeted molecular pathways with different cargo associated with tumorigenesis [81]. Based on molecular investigations, genomics (miRNAs, lncRNAs, etc.) and several factors can initiate the mechanisms associated with carcinogenesis inside the cancer cells (Table 2). Inside the tumor parenchyma, TME with specific physicochemical properties exists for the regulation of cancer cell dynamic growth [82]. TME is composed of heterogeneous cells (tumor cells, stromal cells, ECs, epithelial cells, MSCs, fibroblasts, and immune cells), ECM components, vascular units, and secretory ingredients [82, 83]. The orchestrated and mutual cross-talk between cancer cells with TME can lead to tumor development, expansion, and metastasis [83]. Commensurate with these comments, the organization, and alignment of TME components are critical to tumor cell function [84]. Whether and how Exos can affect the physicochemical properties of TME, non-cancer cells, and cancer cells is at the center of the debate. Emerging data have indicated that Exos can educate cells inside the tumor parenchyma and alter the physicochemical properties of TME. Tumor cell-derived Exos can change the function of TME cells and vice versa. The mutual interaction between the cancer cells and non-cancer stromal cells can dynamically alter the physicochemical properties of TME [79]. Noteworthy, inside the solid tumor parenchyma, the existence of hypoxic conditions increases the local levels of lactic acid, and ECM acidosis [85]. Under such conditions, cancer cells can exhibit rapid proliferation by engaging a mechanism that is so-called metabolic reprogramming [86]. Exos can increase the resistance of vulnerable cancer cells and non-cancer stromal cells to lower pH values via the transfer of mitochondrial particles to restore the production of ATP in these cells [80]. Unlike solid tumors, TME is different in hematologic cancers. Tumor cells can interact with the bone marrow microenvironment and prolonged interaction can lead to the acquisition of a cancerous niche [87]. The role of Exos in the progression of leukemia, invasion, angiogenesis, and inhibition of hematopoiesis has been addressed [88]. Under hypoxic conditions, Exos can foster tumorigenic properties via the regulation of EMT, invasion, survival rate, and maintenance of stemness features. Molecular analyses have confirmed that the density of hypoxia-Inducible factor-1 alpha (HIF-1α) is high in hypoxic cancer cell Exos [82]. Exos can alter the number of TME cells like T lymphocytes, NK cells, T regulatory lymphocytes, dendritic cells (DCs), MSCs, ECs, and MDSCs [89]. In a study conducted by Hou et al., they found that chondrosarcoma cell Exos promote the polarization of macrophages towards M2 type in response to hypoxia, ultimately leading to enhanced metastasis rate [90]. In a similar experiment, it was shown that hypoxic lung cancer Exos with luminal miRNA-21 affects IRF1 and increases M2 type macrophages [91]. It is thought that hypoxic conditions can alter the cargo type, biogenesis and secretion of Exos from cancer cells [92]. The levels of ceramides are increased by the activity of ceramide enzymes in response to hypoxia [93]. Of note, the type of molecules sequestrated into hypoxic ILVs is also changed compared to the normoxic conditions. Along with the expression of HIF-1α, miRNA-210, -21-3p, 125b-5p, 181d-5p levels are increased in released Exos in a HIF-1α-dependent manner [94–96]. Interestingly, the size of Exos is reduced under hypoxic conditions because the lack of coordination between the different parts of endosomal system [97]. Taken together, hypoxia is influencing factor in invasion, and metastasis of tumors toward remote site via the release of Exos with specific cargo from host cancer cells.

**Table 2:** Exosome cargoes effective on the promotion of cancers

	**Biomarker**	**Source**	**Molecular level effect**	**Phenotypic effect**	**Reference**
**miR**	miR-519a-3p	Gastric Cancer	MAPK/ERK pathway	Angiogenesis & Metastasis	[98]
	miR-934	Colorectal Cancer	PTEN/ PI3K/AKT	Metastasis	[99]
	miR-146a	Breast Cancer	Wnt/βCatenin	Invasion & Metastasis	[100]
	miR-128-3p	Colorectal Cancer	TGF-β/SMAD & Jak/STAT3	EMT	[101]
	miR-3473b	lung cancer	NF-κB	colonization	[102]
	miR-345-5p	Colorectal related CAFs	CDKN1A	tumor cell progression & metastasis	[103]
	miR-20a-5p	CAFs	Wnt/β-catenin	promotes hepatocellular carcinoma	[104]
	miR-221-3p	M2-MQ	SOCS3/JAK2/STAT3	osteosarcoma metastasis	[105]
	miR193a-5p	TAM	TIMP2	renal cancer progression	[106]
	miR-3157-3p	non-small cell lung cancer	TIMP/KLF2	angiogenesis	[107]
**lncRNA**	SNHG16	breast cancer	TGFβ/SMAD	induction of CD73 + γδ1 Tregs	[108]
	SNHG10	colorectal cancer	INHBC & TGFβ	inhibit cytotoxicity in NKCs	[109]
	SNHG1	Hypoxic breast cancer	Jak2	proliferation & angiogenesis	[110]
	PARTI	Esophageal cancer	miR302a-3p/CDC25A	angiogenesis	[111]
	CDKN2B-AS1	thyroid cancer	miR-122-5p/ P4HA1	Migration & Invasion	[112]
	TTN-AS1	gastric cancer	miR-499a-5p/ZEB1/CDX2	growth & metastasis	[113]
	AP000439.2	clear cell renal cell carcinoma	STAT3	tumor progression	[114]
	PCAT1	Colorectal Cancer	miR-329-3p/Netrin-1-CD146	Metastasis	[115]
	LINC00313	non-small cell lung cancer	miR-135a-3p/STAT6	M2-MQ differentiation	[116]
	NEAT1	Hepatoblastoma	miR-132/MMP9	induce BMSCs to myofibroblasts	[117]
**circRNA**	circ_FMN2	Colorectal cancer	miR-338-3p/MSI1	Cancer progression	[118]
	circPACRGL	Colorectal cancer	miR-142-3p/miR-506 3p-TGF-β1	Cancer progression	[119]
	circCCAR1	Hepatocellular carcinoma	miR-127-5p/WTAP	dysfunction of CD8 + T cells	[120]
	circRNA100338	Hepatocellular Carcinoma	-	Promote metastasis	[121]
	circTGFBR2	Hepatocellular Carcinoma	miR-205-5p/ATG5	Cancer progression	[122]
	circDennd1b	Pituitary Adenoma	miR-145 5p/ONECUT2	Cancer progression	[123]
	circ_0051799	Lung adenocarcinoma	miR-214-3p/IGF2BP3 /JAK/STAT	Cancer proliferation and metastasis	[124]
	circ_0005615	Colorectal cancer	miR-873-5p/FOSL2	Cancer progression	[125]
	hsa_circ*IFNGR2*	Ovarian cancer	miR-378/ST5	Metastasis	[126]
**Protein**	DNAJB11	Pancreatic ductal adenocarcinoma	EGFR/MAPK	Cancer development	[127]
	B7-H3 (CD276)	Colorectal cancer	AKT1/mTOR/VEGFA	Angiogenesis & Metastasis	[128]
	DPP4	Colon Cancer	Twist1/Smad	Angiogenesis	[129]
	ENO1	Hepatocellular Carcinoma	FAK/Src-p38MAPK & integrin α6β4	Cancer growth and metastasis	[130]
	CD44	Gastric Cancer	YAP/CPT1A	Metastasis	[131]
	GDF15	Colorectal cancer	Bcl-2/caspase-3	Muscle atrophy	[132]
	ITGB1	Rectal cancer	NFκB	Activation of lung fibroblasts	[133]
	MLF1	Intrahepatic cholangiocarcinoa	EGFR/AKT & Wnt/β-catenin	tumor cells’ proliferation and metastasis	[134]
	MUC13	Esophageal cancer	GLANT14, MUC3A, MUC1, MUC12, and MUC4/ O-glycan process	Cancer development	[135]
	RNF126	Nasopharyngeal carcinoma	PTEN/PI3K/AKT	Cancer growth and metastasis	[136]

*Abbreviation*: *miR* Micro-RNA, *MAPK* Mitogen-activated protein kinase, *ERK* Extracellular signal-regulated kinase, *PTEN* Phosphatase and tensin homolog, *PI3K* Phosphoinositide 3-kinases, *AKT* Protein kinase B, *WNT* Wingless-related integration site, *TGF-β* Transforming growth factor-β, *SMAD* Suppressor of Mothers against Decapentaplegic, *JAK* Janus kinases, *STAT* Signal transducers and activators of transcription, *NF-κB* Nuclear factor kappa-light-chain-enhancer of activated B cells, *CDKN1A* Cyclin dependent kinase inhibitor 1A, *SOCS* Suppressor of cytokine signaling, *TIMP* Tissue inhibitor of metalloproteinases, *KLF* Kruppel-like factor, *INHBC* Inhibin subunit betac, *CDC25A* Cell division cycle 25A, *P4HA1* Prolyl 4-hydroxylase subunit alpha 1, *ZEB1* Zinc finger E-box binding homeobox 1, *CDX2* caudal type homeobox 2, *MMP9* Matrix metallopeptidase 9, *MSI1* Musashi RNA binding protein 1, *WTAP* WT1 associated protein, *ATG5* Autophagy related 5, *ONECUT2* One cut homeobox 2, *IGF2BP3* Insulin like growth factor 2 mRNA binding protein 3, *FOS* Like 2, *ST5* Suppression of tumorigenicity 5, *EGFR* Epidermal growth factor receptor, *mTOR* Mammalian target of rapamycin, *VEGFA* Vascular endothelial growth factor A, *FAK* Focal adhesion kinase, *YAP1* Yes1 associated transcriptional regulator, *CPT1A* Carnitine palmitoyltransferase 1A, *BCL-2* B-cell lymphoma 2, *MUC* Mucin

#### Fibroblasts and other tumor-associated cells

**Fig. 2:**
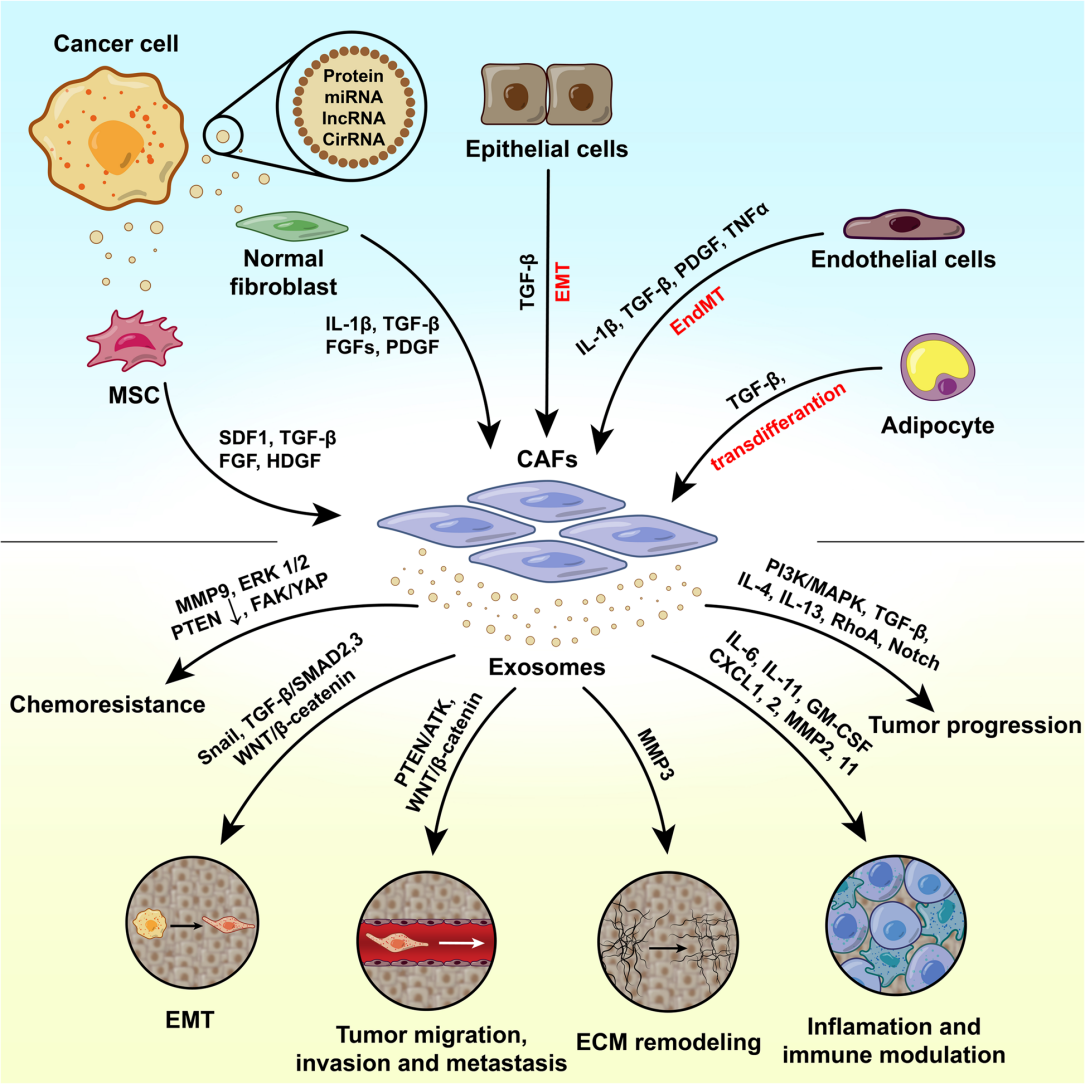
Underlying mechanisms associated with the generation of CAFs. These cells can regulate several cancer resistance mechanisms in a paracrine manner via the release of Exos with certain cargoes. Abbreviations: Notch: Neurogenic locus notch homolog protein, TGF-β: Transforming growth factor-β, IL-6: Interleukin 6 WNT: Wingless-related integration site, HIF-1α: Hypoxia-inducible Factor 1α, NF-κB: Nuclear factor kappa-light-chain-enhancer of activated B cells, Snail: Zinc finger protein SNAI1, Slug: Zinc-Finger Protein Slug, Zeb: Zinc finger E-box-binding homeobox

Cancer-associated fibroblasts (CAFs) are specific fibroblast types within the TME in several tumors [137]. CAFs do not solely originate from activated tumor fibroblasts. Different cells inside the TME such as MSCs, monocytes, adipocytes, smooth muscle cells, pericytes, and CSCs can commit into CAFs [138]. This biological activity is promoted by mechanisms called epithelial-mesenchymal transition (EMT) and endothelial-mesenchymal transition (EndMT) [139]. The process of transformation of normal fibroblasts to CAFs is stimulated via the modulation of several signaling pathways like transforming growth factor beta (TGFβ1)/suppressor of mothers against decapentaplegic (SMAD), stromal-derived factor 1 alpha (SDF-1α)/C-X-C chemokine receptor type 4 (CXCR4), IL-1β/NF-κB, IL-6/JAK/ ROCK/STAT3, Wnt, and HIF-1α. Depending on the malignancy rate and type of cancer, specific signaling pathways can be involved in the production of CAFs from normal fibroblasts [140, 141] (Fig. 2).

It is postulated that the activity of non-cancer stromal cells is controlled by the CAFs. CAFs can regulate tumor cell proliferation, resistance to chemotherapeutics, metastasis, and apoptotic changes [140, 142–144]. Studies have shown that the activity of factors associated with Exo biogenesis in CAFs is higher as compared to normal fibroblasts [145]. CAF-derived Exos can regulate cancer cell proliferation, vascularization, and blood supply for tumor niches [146]. In ovarian cancers, CAFs produce Exos with low-levels of miR-29c-3p and metastatic behavior [137]. As such, CAFs can control the progression and expansion of colorectal cancer via the alteration of CDKN1A and SNX2 signaling pathways via exosomal miR-345-5p [103, 142]. The existence of miR-345-5p in CAF Exos can down-regulated LIMA1 leading to the activation of the Wnt/β-catenin pathway and hepatic carcinoma cell proliferation [104]. CAFs can also change the metabolism of cancer cells via the production of Exos with specific cargoes. For instance, exosomal lncRNA, namely LINC01614, stimulates the metabolism of glutamine, and thus cancer cell function is dependent on this amino acid [147]. In another work done by Yang and co-workers, CAF exosomal circular RNA, named circEIF3K, increased colorectal cancer progression in a hypoxia-dependent manner via the modulation of miR-214/PDL1 [148]. Like circEIF3K, CAFs can release Exos with other circular RNAs such as circZFR with the potential to alter Stat3/NF-κB molecular pathway and enhance hepatocellular carcinoma cancer growth and resistance to chemotherapy [149].

#### TME cells

MSCs are TME cellular components with self-renewal and multi-lineage differentiation capacity [150]. Although the immune-modulatory properties of MSCs have been previously addressed [81], MSCs participate in TME remodeling via the production of Exos [150]. For example, MSC Exos can induce angiogenesis, proliferation, apoptosis, metastasis, dormancy, drug resistance, and immune cell suppression via the alteration of certain effectors such as mTOR, AKT, PKC, MAPK, JNK, p53, NFE2L2 and ERK1/2 [150–153]. Of course, the function and tumorigenic behavior of MSCs within the TME can be regulated in a paracrine manner via cancer cell Exos. In a study conducted by Gyukity-Sebestyén et al., they claimed that melanoma cell Exos up-regulate PD-1 and phenotype acquisition of MSCs, leading to increased cell survival signals and tumor progression [81]. The active and mutual cross-talk between bone marrow MSCs and tumor cells can result in the progression of leukemia [154, 155]. Of course, it should not be forgotten that MSC Exos can also exert tumoricidal effects. How and by which mechanisms the tumorigenic and/or tumoricidal properties of MSC Exos are prominent needs further investigation.

**Fig. 3:**
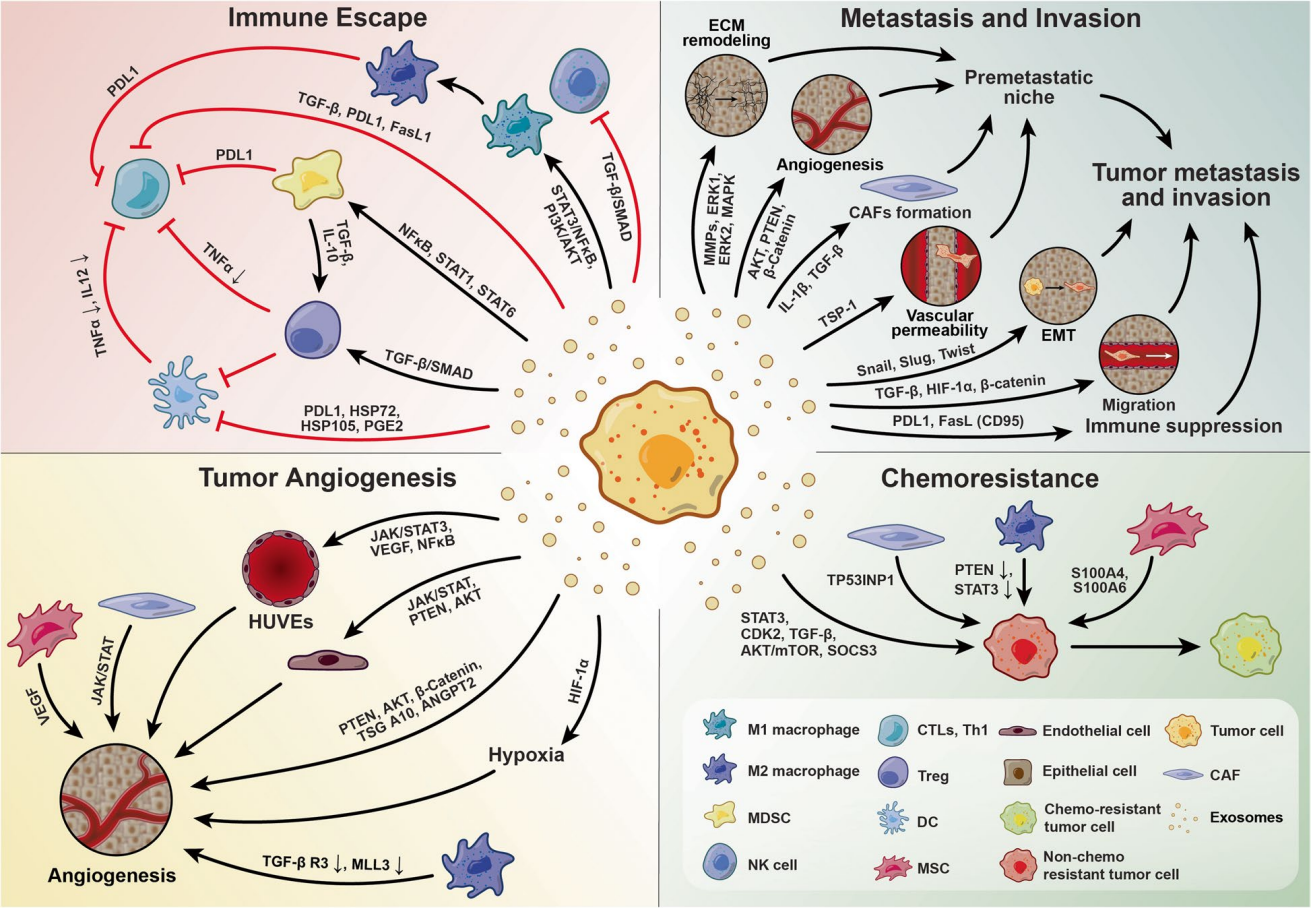
Cancer cells can use Exos for the regulation of various signaling factors associated with tumor metastatic behavior, chemoresistance, vascularization, and immune escape. Abbreviations: CAFs: Cancer-associated fibroblasts, TGF-β: Transforming growth factor-β, IL-1 β: Interleukin 1 β, FGF: Fibroblast Growth Factor, PDGF: Platelet-derived growth factor, TNF-α: Tumor Necrosis Factor-alpha, PI3K: Phosphoinositide 3-kinases, MAPK: Mitogen-activated protein kinase, RhoA: Ras Homolog Family Member A, Notch: Neurogenic locus notch homolog protein, IL: Interleukin, GM-CSF: Granulocyte–macrophage colony-stimulating factor, CXCL: CXC motif chemokine ligand, MMP: Matrix metallopeptidase, PTEN: Phosphatase and tensin homolog, AKT: Protein kinase B, WNT: Wingless-related integration site, Snail: Zinc finger protein SNAI1, SMAD2: SMAD family member 2, SMAD3: SMAD family member 3, ERK: Extracellular signal-regulated kinase, FAK: Focal adhesion kinase, YAP: Yes-associated protein 1, SDF1: Stromal cell-derived factor 1, HDGF: Hepatoma-derived growth factor, EMT: Epithelial-mesenchymal transition, EndMT: Endothelial-mesenchymal transition, ECM: Extracellular Matrix, MSC: Mesenchymal stem cells

Different mechanisms are involved in immunity against tumor cells along with the activity of natural killer (NK) cells [156]. Tumor-associated antigens are captured by antigen-presenting cells (APCs) like macrophages, T lymphocytes, etc. and further presentation of these antigens to effector immune cells results in tumor cell cytotoxicity [157, 158]. It has been elucidated that TME Exos can reduce the function of immune cells such as NK cells, DCs, and B and T lymphocytes via the regulation of TGF-β TGF I β-6, TNF-α, CTLA4, PD1 [158]. Under these conditions, Exos can increase the polarization of macrophages toward the M2 type [159, 160]. It has been indicated that macrophages have a dual function inside the TME. The M1 macrophages exhibit tumoricidal effects while M2 macrophages can help the tumor cells to proliferate and metastasize [83]. Within the TME, the largest fraction of macrophages is the M2 type while in the early stages of tumor formation, M1 macrophages are dominant and they commit to the M2 type over time [161]. This phenomenon is promoted in part via the production of IL-6-loaded Exos via cancer cells that dictate specific phenotypes for tumor-associated macrophages (TAMs) [162, 163]. Such mechanism has been indicated in pancreatic cancer cell Exos. These Exos harbor FGD5-AS1 and IL-6 with the potential to increase tumor cell metastasis and survival via the promotion of M2 TAMs via the STAT3/NF-κB pathway [162]. M2 TAM Exos with lncMMPA can increase the glucose metabolism within the TME of hepatocellular carcinoma [163]. M2 TAM Exosomal miR-221-3p can increase osteosarcoma cell metastasis via the modulation of the SOCS3/JAK2/STAT3 pathway [105]. In line with the induction of tumor cell metastasis and proliferation, M2 TAM Exos can increase vasculogenesis, known also vasculogenic mimicry (VM), within the tumor parenchyma by increasing vascular density and blood supply. M2 TAM Exos containing miR193a-5p can increase tumor progression in VM-dependent mechanisms via the TIMP2 pathway [106] (Fig. 3).

Despite the tumoricidal properties of T lymphocytes, TME Exos can suppress the activity of these cells against tumor cells via the transfer of several signaling molecules such as miRNAs, circular RNAs, lncRNAs, TGF-β, PDL1, and PGE_2_. TGF-β can inhibit the commitment toward Th1 and Th17 phenotypes. Exosomal miRNA, PDL1, and TGF-β induce the activity of T_reg_ lymphocytes. On the other hand, Exos can stimulate T cell apoptosis and exhaustion via FasL, TRAIL, TIM3, LAG3, and miRNA [13, 120]. Exosomal PGE2, CD39, and CD73 can alter the metabolic state of T cells, and the function of T lymphocytes is inhibited indirectly via PDL1, TGFβ, and PGE_2_ after suppression of DCs [164]. Hepatocellular carcinoma Exos with circCCAR1 can promote inactivation of CD8^+^ lymphocytes via the stimulation of PDL1 [120]. Along with these changes, the phosphorylation of hepatocyte growth factor receptor substrate (HRS) can limit the recruitment of CD8^+^ lymphocytes [165]. The increase of the Th17 subset within the tumor niche is related to tumor mass expansion. The release of Exos containing lncRNA CRNDE-h from colorectal cancer cells promotes the number of Th17 cells and thus cancer mass development [166]. Tumor cell Exo miR-208b and SNHG16 can affect the function of T_reg_ lymphocytes and DCs within the TME. Along with these changes, the number of recruited CD4^+^ T lymphocytes and local IFN-γ is reduced [83]. These miRNAs can increase the number of CD73 + γδ1 T_reg_ lymphocytes via the modulation of PDCD4 and TGFβ/SMAD pathways [108, 167] (Fig. 3).

Like T lymphocytes, the critical roles of NK cells should not be neglected in different malignancies. These cells and frontline cells promote tumoricidal effects via functional receptors [13]. The physical contact of NK cells with tumor cells leads to whole-cell lysis although the production of various cytokines can affect the anti-tumor activity of MK cells [158, 168]. Like other non-cancer stromal cells, tumor cell Exos can impair the function of NK cells via stimulation/inhibition of specific receptors within the cancerous niche, resulting in anti-tumor activity suppression [13]. In this scenario, hepatocellular carcinoma cells can decrease the local contents of IFN-γ and TNF-α via exosomal circUHRF1 and thus NK cell activity [169]. The stimulation of NK cell TGFβ/SMAD pathway by renal cell carcinoma Exos decreases the anti-tumor sensitivity following NKG2D suppression and induces tumor immune escape [83, 158, 170]. It was suggested that the attachment of certain exosomal factors such as ProNGF and Sortilin to surface receptor p75NTR increases the apoptotic changes in NK cells within the parenchyma of lung tissue cancers [170]. Likewise, colorectal cancer Exos with lncRNA SNHG10 can increase the NK cytotoxicity via up-regulation of INHBC from the TGF-β pathway [109]. The interaction of exosomal miRNA-221-5p and miRNA-186-5p with certain mRNAs (DAP10, and CD96), and perforin genes has been approved in bladder cancers [158]. Along with these comments, the TGF-β signaling pathway is one of the main targets for tumor cell Exos to control the activity of NK cells. In support of this notion, acute lymphocytic leukemia cell Exos can diminish the anti-tumor activity, proliferation, cytotoxicity, and inhibition of cytotoxic granules of NK cells via the TGF-β signaling pathway [168] (Fig. 3).

MDSCs are heterogeneous and immature bone marrow progenitor cells with morphologies similar to neutrophils and monocytes [89, 171, 172]. It is suggested that MDSCs can be committed into M1 and M2 macrophages [173]. The dynamic growth and differentiation of MDSCs in TME are regulated by several cytokines such as G-CSF, M-CSF, SCF, VEGF, and unsaturated fatty acids, IFN-γ, IL-1β, TNF-α, IL-4, -6, -13 by the modulation of NF-κB, STAT1, and STAT6 signaling pathways [174]. The activity of MDSCs can lead to suppression of CD8^+^ lymphocytes, stimulation of T_reg_ cells, increase of Th17 lymphocytes, orientation of macrophages toward M2 type, and inhibition of B lymphocytes and NK cells [171, 172]. MDSC Exos harbor several factors (S100A8/A9, HSP72, CD47, TSP1, TGF-β, and PGE_2_), miRNAs (miRNA-21, -9, and -181a) to target certain signaling molecules such as STAT3, RORα, SOCS3 and PIAS3 inside the immune cells [83, 172, 175]. In response to exosomal miRNA-21, and miRNA-29a, MDSCs can promote the growth of tumor cells after the modulation of ROR-A/PTEN and Prkar1α signaling pathways [89]. Noteworthy, CAF exosomal miR-21 and IL-6 can increase the differentiation of MDSCS toward monocyte-macrophage lineage via the modulation of STAT3 [25, 172] (Fig. 3).

#### Effects of Exos on tumor cell invasion and metastasis

The metastasis is a complex biological phenomenon with sequential steps that help the tumor cells to separate from primary sites and migrate to close and remote sites. Upon reaching the new microenvironments, these cells can proliferate and produce ectopic foci [176]. To increase the possibility of metastasis, the suppression of immune cells and cancer cell resistance are critical features for the development of a pre-metastatic niche. As above-mentioned T_reg_ lymphocytes are the main cell elements in the promotion of tumor cell metastasis to remote sites [177]. Emerging data confirmed the influence of tumor cell Exos in the formation of the pre-metastatic niches. In terms of dynamic trafficking, it should be noted that Exos can be easily distributed inside TME and separate from each other due to net negative charge at their surface [80]. These features mitigate in situ Exo agglutination inside the TME and increase the transfer into remote sites. On the other hand, certain cargo types potentiate Exos to alter the physicochemical behavior of TME, and metastatic behavior of tumor cells via the alteration of targeted signaling pathways, induction of angiogenesis, and immune cell suppression [178]. Some tumor cells are supposed to pass the tissue natural barrier, *i.e*. blood–brain barrier (BBB), and lung-blood barrier, in addition to blood–tumor barrier (BTB) [179, 180]. Due to specific physicochemical properties, and the existence of certain ligands (integrins) and internalization mechanisms, Exos can, in part, circumvent these obstacles and transfer the cytokines, and growth factors into the TME and remote sites. Exos can change the composition of ECM by the alteration of specific molecular pathways in favor of tumor cell survival and proliferation [180, 181]. Of note, the type and amount of exosomal integrins can pre-determine the on-target tissues and place of metastatic foci [84]. Tumor cell Exos exhibit the prominent capacity to cross the BBB interface. For instance, the transfer of lung cancer cell Exos from BBB increases the apoptotic astrocytes inside the brain parenchyma. Besides, because of specific immunosuppressive agents and inflammatory cytokines, these Exos can prepare the brain microenvironment for the development of metastatic sites [179] (Fig. 3).

Like tumor cells, CAFs actively participate in the formation of pre-metastatic via the release of several chemokines, growth factors, synthesis of certain ECM components, and matrix metalloproteinases (MMPs) [182]. Colorectal cancer cells produce HSPC111 (c-Myc target gene)-enriched Exos that facilitate the development of metastatic foci in other tissues by the change of lipid metabolism [178]. CAF Exos can reduce the activity of the mitochondrial electron transport chain and induce the glycolysis pathway in tumor cells, making these cells resistant to a lack of O_2_ and nutrients [183]. To increase the metastatic behavior of tumor cells, their migration capacity should be stimulated. Tumor cell Exos with specific cytokines TGF-β, HIF-1α, β-Catenin, and Caveolin-1 can increase the motility of neighboring cells within the TME [184]. Upon reaching the target sites, migrating tumor cells hide and undergo dormancy. Dormant tumor cells educate the resident immune cells to acquire tumor-supporting phenotype to mimic pre-metastatic TME [79]. It was well-established that Exos can affect the dormant tumor cells and their subsequent biological properties [185]. At the primary site, Exos can weaken intercellular communication via the disassociation of adhesion molecules. For example, colorectal tumor cell Exos with luminal ADAM-17 content disassociates E-cadherin in juxtaposed cells and loosens cell-to-cell attachment, leading to enhanced tumor cell migration and the possibility of metastasis to hepatic tissue [176]. The loss of vascular EC-to-EC connection is thought to be another mechanism for the metastasis of tumor cells to remote sites. Exosomal miRNAs such as miR-105 produced by breast tumor cells weaken the tight junction of vascular cells and diminish the integrity of basal membrane, leading to the permeability of blood and lymphatic vessel and increase of metastasis to remote sites [79, 83].

**Fig. 4:**
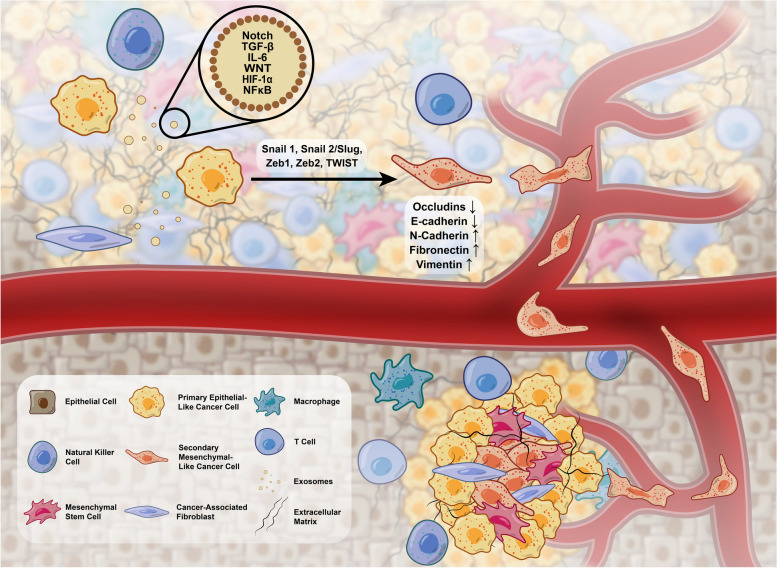
Oncogenic properties of Exos. Exos can transfer specific signaling molecules with the potential to increase tumor cell metastasis and the formation of metastatic foci in remote sites. Abbreviations: MMPs: Matrix metallopeptidase, ERK: Extracellular signal-regulated kinase, MAPK: Mitogen-activated protein kinase, PTEN: Phosphatase and tensin homolog, AKT: Protein kinase B, TGF-β: Transforming growth factor-β, IL-1 β: Interleukin 1 β, TSP-1: Thrombospondin-1, Snail: Zinc finger protein SNAI1, Slug: Zinc-Finger Protein Slug, HIF-1α: Hypoxia-inducible Factor 1α, PDL1: Programmed death-ligand 1, FasL: Fas ligand, SMAD:, PI3K: Phosphoinositide 3-kinases, STAT: Signal transducers and activators of transcription, JAK: Janus kinases, NF-κB: Nuclear factor kappa-light-chain-enhancer of activated B cells, HSP: Heat shock proteins, PGE2: Prostaglandin E2, IL: Interleukin, TNFα: Tumour Necrosis Factor alpha, VEGF: Vascular endothelial growth factor, TSG A10: Testis-specific gene antigen 10, ANGPT2: Angiopoietin-2, TGF-βR3: Transforming growth factor-β receptor 3, S100A4:, TP53INP1: Tumor Protein P53 inducible nuclear Protein 1, mTOR: Mammalian target of rapamycin, SOCS3: Suppressor of cytokine signaling 3, EMT: Epithelial-mesenchymal transition, ECM: Extracellular Matrix, CAF: Cancer Associated fibroblast, DCs: Dendritic cells, Treg: Regulatory T cell, Th1: Type 1 T helper, Th17: Type 17 T helper, CTLs: Cytotoxic T lymphocytes, MDSCs: Myeloid-derived suppressor cells, MSC: Mesenchymal stem cells, NK: Natural killer cells

The modulation of EMT and balance between the epithelial and mesenchymal phenotypes is another mechanism in the development of the pre-metastatic niche. By the promotion of EMT, the levels of E-cadherin are reduced while the cellular content of vimentin, N-cadherin, and fibronectin is increased. Besides to induction of cell resistance to apoptotic changes, these features weaken the connection of tumor cells with the underlying basal membrane and increase the possibility of metastasis [186, 187]. CAF and tumor cell Exos with specific cargo types can stimulate the process of EMT via targeting certain effectors Snail, Slug, Zeb1/2, Twist, etc. [186, 188, 189]. Along with EMT, the stimulation of EndMT and differentiation of CSCs into ECs has been indicated by CAF Exos that lead to blood supply into the TME and metastasis [190] (Fig. 4).

#### Exos and tumor angiogenesis

Angiogenesis is the process of de novo blood vessels from parent vessels to support tumor cell survival, growth, and metastasis [85]. It has been shown that Exos can harbor pro-angiogenesis factors and stimulate TME vascularization [85, 110]. Exos can affect specific effectors associated with angiogenesis such as Akt, PTEN, β-Catenin, TSGA10, and ANGPT2 [85]. The uptake of colorectal tumor cell Exos containing B7-H3 molecule by human ECs led to tubulogenic behavior via the activation of Akt/mTOR and the VEGFA molecular pathways [128]. Likewise, lung cancer cell miR-3157-3p-enriched Exos up-regulates VEGF, MMP2, and 9 and Occludin [107]. It is believed that hypoxic tumor cells can produce Exos with angiogenic potential [107]. Prolonged hypoxic conditions increase the accumulation of HIF-1α and angiogenesis via the release of exosomal Wnt4a and activation of β-Catenin [83, 85]. In light of hypoxia, the Exos with higher levels of lncRNA SNHG1 and mir-216b-5p are released form breast cancer cells and the uptake of these Exos promotes angiogenesis in human ECs via Janus kinase 2 (JAK2) [110]. In a similar study, data confirmed that hypoxic pancreatic cancer cells produce Exos with high levels of miR-30b-5p. This factor can stimulate angiogenesis via the inhibition of Gap Junction Protein Alpha 1 (GJA1) [191]. Likewise, thyroid cancer cells can control the angiogenesis in a paracrine manner via the release of Exos enriched in lncRNA FGD5-AS1. This factor targets miR-6838-5p and VAV2 related to actin re-organization and cytoskeletal remodeling [192]. Along with the direct effect of tumor cell Exos and endothelial lineage, the uptake of these nanoparticles by M2-type macrophages can lead to the promotion of angiogenesis. It has been found that tumor cell Exos can recruit neutrophils and increase M2-type polarization of macrophages to support ECs [79] (Fig. 3).

#### Exos and tumor cell resistance

Chemo-resistance is one of the major challenges that reduce the efficiency of therapeutic protocols [23]. Exos with specific cargoes [P-gp, Survivin, DNMT1, Annexin A3, ATP7A, ATP7B, MRP1, p-STAT3) and miRNAs (miRNA-222-3p, -214, -100-5p, -567, -155-3p, -21, -433, -21-3p, -1246, -223, -365, -19b, -20a, -32-5p, -501, -447-5p, -99a-5p, -125b-5p, -210 & and -155] can increase tumor cell resistance via engaging different mechanisms such as DNA repair, apoptosis inhibition, alteration of drug targets, and efflux, up-regulation of MDR and oncogenes, down-regulating of tumor suppressor genes, EMT induction, autophagy stimulation [23, 193]. The transfer of Exos from resistant cells to sensitive cells is an effective way to treatment failure. In this regard, CAF Exos can educate the neighboring cells to resist chemotherapeutics [194]. Of note, in response to chemotherapy, tumor cells produce Exos containing ANXA6 that induces stemness phenotype in cancer cells via the regulation of ONECUT2. Along with these changes, exosomal levels of miR-378a-3p and miR-378d are increased in breast tumor cells after chemotherapy, resulting in cancer resistance via the EZH2/STAT3 pathway [26]. It seems that the levels of resistance factors are higher in Exos from resistant tumor cells compared to non-resistant counterparts. Tamoxifen-resistant breast tumor cells release Exos with higher luminal miRNA-205 which increases resistance to these drugs in other cells by targeting E2F1 [26]. In a similar work, it was indicated that doxorubicin-resistant neuroblastoma cells with prominent glycolysis activity produce Exos with higher circDLGAP4 contents that induce resistance in sensitive cells by targeting Hexokinase 2 [41].

As mentioned earlier, the reduction of therapeutic agents inside the tumor cells is another anti-tumoricidal property [23]. In this scenario, tumor cells can eliminate internalized chemotherapeutics via the activation of transport pumps. It is suggested that ABC transmembrane transporters (ABCB1, P-gp, MDR1, ABCCs, ABCG2, and MXR) can contribute to the efflux of various drugs from tumor cells [195]. Exos can regulate the expression and activity of cell membrane transporters. For instance, Exos containing P-gp promotes the transfer of drug resistance in recipient tumor cells. MSC Exos with miR-301-3p stimulates multidrug resistance of gastric tumor cells by inhibiting thioredoxin-interacting protein TXNIP [31]. Immune escape, angiogenesis, and the creation of CAFs are other mechanisms associated with tumor cell resistant [23]. As above-mentioned, MDSCs increase the chemo-resistance of tumor cells by different mechanisms, such as inhibition of macrophage polarization towards M1 type, promotion of angiogenesis, interaction with IL6, and increasing the secretion of S100A8/A9 [172]. The transfer of specific factors from CAFs to tumor cells makes them cells resistant to chemotherapeutics. Following gemcitabine treatment, pancreatic ductal adenocarcinoma CAFs can internalize the Exos with ACLS4, followed by induction of gemcitabine resistance in cancer cells via miR-3173-5p [29]. Besides, the transfer of MMP-14 via Exos from resistant pancreatic ductal adenocarcinoma cells to sensitive tumor cells increases their survival [47]. A recent study showed that acute myeloid leukemia cell Exos induce drug resistance by upregulating S100A4 (calcium-binding protein) in other cells [196]. The critical role of exosomal miR-21-5b and S100A6 has been documented in other tumor cell types [30] (Fig. 3).

### Anti-oncogenic properties Exos

Besides their oncogenic roles, Exos can exert inhibitory effects on inhibiting tumor cell growth, progression, migration, and invasion via genetic cargo with tumor-suppressing capacities like miRNAs, pro-apoptotic factors, and anti-inflammatory cytokines [197]. In contrast to the resistant tumor cells and CSCs Exos, normal cells and non-resistant cancer cell Exos can expedite the immune system reactivity and anti-tumor properties [198]. The activation of DCs by hepatocellular carcinoma cell Exos increase the number of recruited T lymphocytes in TME with simultaneous elevation of IFN-γ. Under such conditions, leukocytosis and increased cytotoxic T lymphocytes (CTLs) are prominent [199]. Interestingly, brain microvascular ECs Exos with high levels of ECRG4 can suppress the inflammation and angiogenesis inside the glioma tumor parenchyma by inhibiting the P38-MAPK signaling pathway [200]. In an experiment conducted by Wang et al., they showed that exosomal miRNA-363-5p can target the PDGFB pathway and can inhibit breast cancer tumor cell proliferation and migration [201]. It was suggested that some tumor-specific antigens (such as Her2/Neo, Mart1, TRP, and gp100) can be transferred by Exos, leading to the promotion of the immune system against cancer cells [202]. Multiple myeloma Exos IL15/IL15R complex can initiate the proliferation and expansion of NK cells. In activated NK cells, the continuous production of IFN-γ occurs via the stimulation of the TLR2/HSP70/NF-κB pathway. To be specific, tumor cell Exos can frustrate NK cells and increase cytolytic and migration properties in an HSP70-dependent manner [203]. The inhibition of PD-1 in CD8^+^ lymphocytes was reported after exposure to miR-15a-5p containing hepatocellular carcinoma cell Exos. PD-1L-expressing tumor cells can easily escape from the immune system [204]. In pulmonary cancer, the release of GPC5 (belonging to heparan sulfate proteoglycan) containing Exos contributes to the reduction of angiogenic potential in lymphatic ECs via suppression of PTK2, and endothelial migration. These features are associated with the expression of the CTDSP1 gene and activation of the AhR-PRNT signaling pathway [205].

#### MSC and immune cell Exos

MSC Exos with various miRNAs and tumor suppressor profiles are suggested biological weapons against several cancer types [151]. In this regard, Xu et al. claimed that bone marrow MSC Exos containing miR-16-5p can inhibit the ITGA2, resulting in the reduction of colorectal cancer cell proliferation, migration, and invasion. Meanwhile, the number of apoptotic tumor cells also increased [206]. In another study, it was indicated that miRNA-let-7c and miRNA-34a containing MSC Exos can effectively reduce the dynamic growth and metastasis of resistant prostate and breast tumor cells, respectively [153, 207]. It seems that several tumorigenic mechanisms can be controlled via MSC Exos in different cell types. Signaling pathways such as LIMK1/Wnt/β-Catenin [208], EMT, TGF-β [209], ZNF367 [17], KLF7/AKT/HIF-1α [210], and Galectin-3 [211] can be modulated via exosomal miRNAs and cargo. These features indicate the anti-tumor activity of MSC Exos with a wide range of functions. Inside the TME, antigen-presenting properties of DCs can be stimulated after exposure to tumor cell Exos. Although DC Exo with notable levels of MHC-I, and -II, CD86, CD80, and HSP can promote T lymphocytes and CD8^+^ cells [14]. Molecular investigations have revealed that the levels of sphingomyelin and phosphatidyl inositol are high in DC Exos, resulting in enhanced stability and circulation time compared to Exo types [212]. The process of antigen presentation from DCs to immune cells is orchestrated via several mechanisms. Naïve DC Exos may be internalized by T lymphocytes or cross-dressed and coated with DC membrane components before uptake by T lymphocytes. Some authorities have documented the internalization of DC Exos by tumor cells and the addition of tumor-specific antigens with stronger immunological properties [213]. Decoration of DC Exos with specific integrin types αMβ2 and ICAM1 can increase the on-target potential effects [212].

**Fig. 5:**
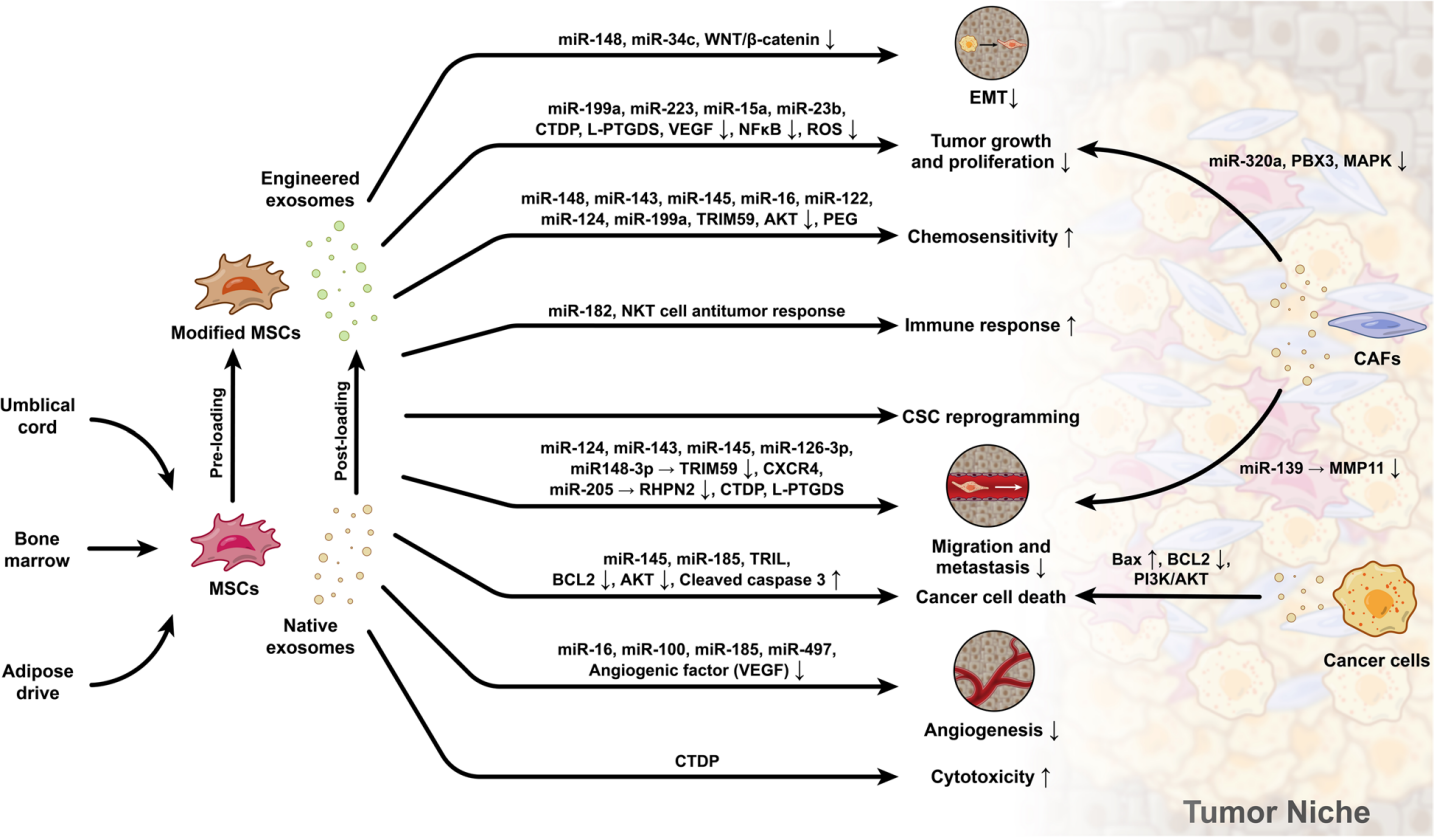
Different stem cell types produce Exos with tumoricidal properties. Abbreviations: MiR: MicroRNA, WNT: Wingless-related integration site, CTDP: Chemotherapy drugs preloaded, AKT: Protein kinase B, TRIM59: Tripartite motif-containing 59, CXCR: C-X-C Motif Chemokine Receptor, PEG: polyethylene glycol, RHPN2: Rhophilin-2, L-PGDS: Lipocalin-type prostaglandin D synthase. TRIL: TLR4 interactor with leucine-rich repeats, BCL-2: B-cell lymphoma 2, VEGF: Vascular Endothelial Growth Factor, BAX: Bcl-2-associated X protein, MMPs: Matrix metalloproteinases, MAPK: Mitogen-activated protein kinase, PBX3: Pre-B-cell leukemia transcription factor 3, CAFs: Cancer-associated fibroblasts, MSCs: Mesenchymal stem cells, CSCs: Cancer stem cells, EMT: Epithelial-mesenchymal transition

Along with DCs, B, and T lymphocytes (CD4^+^ and CD8^+^ subsets) exhibit anti-tumor activities [203]. T cell Exos, especially CD8^+^ lymphocyte Exos, are potent destructive agents after activation by DCs. The Exos can directly attack tumor cells, eliminate TME MSCs, and activate other T lymphocytes. The inhibition of PDL-1 on the surface of tumor cells is also done via the release of PD-1^+^ Exos via specific miRNAs such as miR-16p [14, 214]. Likewise, NK cell Exos can exert tumoricidal effects via the stimulation of apoptosis-related factors such as certain Caspases [160, 215]. The existence of HSP70, and granzyme B in NK cell Exos increases the possibility of apoptosis in tumor cells [203]. NK cell and M1 macrophage Exos with specific cargo, miR-30-3p, and miR-16-5p respectively can reduce the proliferation and invasion of esophageal squamous carcinoma cells and gastric tumor cells via the modulation of PD-L1 [216, 217] (Fig. 5).

## Application of stem cell Exos in cancer therapy

### MSC Exos

Exos can be isolated from MSCs of different tissues [218]. Of note, there are controversies in the application of MSCs for cancer therapy purposes. On one hand, MSCs increase TME remodeling and can foster tumor cell dynamic growth, metastasis, and EMT via the suppression of immune system function [219]. On the other hand, various MSC anti-tumor properties have been shown in several in vitro and in vivo conditions [179, 220, 221]. Data confirmed that MSC Exos possess appropriate biocompatibility, healing capacity, and low-rate toxicity, making them valid tools for therapeutic purposes. The tumoricidal properties of MSC Exos are associated with immune system function, regulation of cell-to-cell interaction, induction of apoptotic changes, inhibition of angiogenesis and tumor cell proliferation, and modulation of drug resistance [222–224]. The anti-angiogenesis potential of MSC Exos in the context of tumor parenchyma leads to the reduction of VEGF, inhibition of NF-κB [225], and mTOR/HIF1A/VEGF axis [226]. The interaction of MSC Exos with CSCs promotes MET, loss of stemness features, and increase of non-CSC phenotype within the parenchyma, resulting in the reduction of tumor cell resistance [227]. In the presence of MSC Exos, NK cells and CD8^+^ T lymphocytes proliferate and these changes coincide with the inhibition of T_reg_ cells and polarization of macrophage to M2 phenotype [228]. As above-mentioned MSC Exos increase the chemo-sensitivity of tumor cells by improving anti-drug resistance. It was found that adipose tissue MSC Exos sensitize breast cancer cells to cisplatin [229]. The combination of photobiomodulation with MSC Exos is suggested as an effective therapeutic protocol in cancer patients [229]. In line with several studies, data have confirmed the eligibility of MSC Exos as valid bioshuttles for delivery of anti-tumor factors, increasing drug sensitivity, and targeted delivery purposes [20]. Compared to transplant cells, the trap of administrated Exos is less in hepatic, splenic, and pulmonary tissues which increases the lifespan, circulation time, and affinity to tumor sites [230]. The tumoricidal properties of umbilical cord MSC Exos have been indicated in cancer of renal, endometrial, and breast tissues [231]. Bone marrow MSC Exos with miRNA-16 can suppress the VEGF factor and thus the angiogenesis and vascular density [232]. Besides, the existence of various anti-tumor factors has been indicated inside these Exos [233]. The anti-tumor potential of MSC Exos is closely associated with cargo type, tissue source, and dose and injection interval. In line with the claim, the anti-tumor properties of umbilical cord MSCs is more than bone marrow MSCs and their Exos [150, 234]. Of course, it should not be forgotten that cancer cell type, malignancy degree, and heterogeneity of TME can affect the function of MSC Exos [235, 236]. In general, the effects of different sources of MSC Exos on various cancers remain unclear (Table 3).

**Table 3:** Application of stem cell exosomes in cancer treatment

Source of SCs-Exo	Intervention/Condition	Type of Cancer	Type of Study/ Animal or cell line	Outcome	Ref
BM‑MSCs-Exos	miR-193a delivery	Lung cancer	In vivo/ Nude BALB/c mice	Suppression of the invasion, migration, and proliferation, reducing cisplatin resistance via downregulating LRRC1	[225]
MSCs-Exos	Injection of menstrual MSCs-Exo into the base of the tumor in a dose-dependent manner	Oral squamous cell carcinoma	In vivo/ Syrian golden hamsters	Inhibition of tumor growth and angiogenesis via VEGF suppression	[237]
MSCs-Exos	miR-143 delivery	Osteosarcoma	In vitro/ Human osteosarcoma cell line 143B	Reduction of the migration	[238]
BM‑MSCs-Exos	Paclitaxel delivery	Breast cancer	In vivo/ Nude BALB/c mice	Inhibition of tumorigenesis	[239]
MSCs-Exos	Intravenous injection of doxorubicin-loaded in MSCs-Exo (DOX@exosome-apt)	Colon cancer	In vivo/ BALB/c mice	Inhibition of tumor growth	[226]
AD-MSCs-Exos	Intravenous injection of miR-199a-loaded in AD-MSCs-Exo	Hepatocellular carcinoma	In vivo/ Nude BALB/c mice	Inhibition of mTOR pathway and improvement of HCC chemosensitivity	[240]
UC-MSCs-Exos	miR-302a-overexpressing in hUCMSC	Endometrial cancer	In vitro/ Human endometrial cancer cell lines ISK and ECC-1	Inhibition of the cyclin D1 expression, suppression of AKT signaling pathway, Inhibition of cell proliferation and migration	[241]
BM‑MSCs-Exos	miR-222-3p delivery	Acute myeloid leukemia (AML)	In vitro/ AML cell line THP-1	Suppression of cell proliferation and promotion of apoptosis by targeting IRF2/INPP4B	[242]
MSCs-Exos	miR-23b-5p delivery	Acute myeloid leukemia (AML)	In vitro/ AML cell line HL-60, THP-1	Reduction of the proliferation and induction of apoptosis via inhibition of TRIM14 and PI3K/AKT pathway	[243]
BM‑MSCs-Exos	Exosome-based dual delivery biosystem for Galectin-9 siRNA and Oxaliplatin	pancreatic ductal adenocarcinoma (PDAC)	In vivo/ Orthotopic PDAC mice	Induction of ICD, reversing the suppressive tumor immune microenvironment, enhancing immunotherapy effectiveness via inhibiting M2 macrophage polarization and the recruitment of cytotoxic T lymphocytes	[244]
UC-MSCs-Exos	miRNA-182 delivery	clear cell renal cell carcinoma (ccRCC)	In vivo/ Orthotopic ccRCC mouse model	Increasing cancer cell death by increasing the NK and T cell proliferation, inhibition of metastasis, and growth of tumors	[245]
MSCs-Exos	TRAIL and Cabazitaxel (CTX) delivery	Oral squamous cell carcinoma	In vivo/ Mouse	Inducing apoptosis by inhibiting the PI3K/Akt/mTOR signaling pathway	[246]
BM‑MSCs-Exos	miR-199a-Cy3 delivery	Glioma	In vivo/ Nude BALB/c mice	Suppressing glioma development and higher sensitivity to temozolomide by inhibiting AGAP2 expression	[229]
AD-MSCs-Exos	pre-transfecting miR-1236 inhibitor into the AD-MSCs and Exo isolation (AD-MSCs-Exo /miR-1236 inhibitor)	Breast cancer	In vitro/ DDP-resistant BC cell lines MCF-7 and MDA-MB-231	Impairing cisplatin (DDP) resistance Through Conveying miR-1236 and suppressing SLC9A1 and the Wnt/β-Catenin Signaling	[247]
BM‑MSCs-Exos	Exosomal circ_0030167	Pancreatic cancer		Reduction of the invasion, migration, and proliferation of PC cells by sponging miR-338-5p and targeting the Wif1/Wnt8/β-catenin axis	[248]
UC-MSCs-Exos	miR-451a and ADAM10 delivery	Hepatocellular carcinoma	In vitro/ Hep3B and SMMC-7721 cell lines	limitation of the EMT of hepatocellular carcinoma (HCC) cells by targeting ADAM10	[249]
BM-MSC-Exos	miR-9-3p delivery	Bladder cancer	In vivo/ BALB/c nude mice	Inhibition of the expression of EMS1, inhibiting the progression of bladder cancer	[250]
UC-MSCs-Exos	miR-424 delivery	Ovarian cancer	In vivo/	Inhibition of the proliferation, migration, invasion, and angiogenesis of ovarian tumors	[19]

*Abbreviation*: *MSCs-Exos* Mesenchymal stem cell-Exosomes, *BM-MSC-Exos* Bone Marrow-mesenchymal stem cell-Exosomes, *UC-MSCs-Exos* Umbilical Cord-mesenchymal stem cell-Exosomes, *AD-MSCs-Exos* Adipose Derived- mesenchymal stem cell-Exosomes, *MiR* MicroRNA, *VEGF* Vascular Endothelial Growth Factor, *AKT* Protein kinase B, *IRF2* Interferon regulatory factor 2, *INPP4B* Inositol polyphosphate 4-phosphatase type II, *TRIM14* Tripartite motif protein, *PI3K* Phosphoinositide 3-kinases, *mTOR* Mammalian target of rapamycin, *ADAM10* A disintegrin and metalloprotease 10, *EMS1* Endothelial cell-specific molecule 1, *ICD* Immunogenic cell death

### CSCs Exos

CSC Exos can be a suitable target for cancer treatment because of their active interaction with TME and control of several mechanisms associated with anaplastic conditions [251]. By sophisticated manipulation, CSC Exos can be used for the disruption of CSCs and non-CSC cancer cells, inhibition of resistance mechanisms, and transmission of stemness features to other cells [252]. The available protocols target certain factors or pathways such as the Notch axis that are eminent in CSCs [253]. Due to distinct physicochemical properties, chemotherapeutics, siRNAs, and immunomodulatory agents can be loaded onto CSC Exos to increase on-target delivery efficiency and reduce off-target side-effects [252, 254]. The conversion of EMT and compelling CSCs to commit to the non-CSC phenotype can lead to tumor cell sensitivity to conventional therapeutic protocols [227]. This approach can be achieved by using certain factors such as all-trans retinoic acid in leukemia cells [255]. The inhibition of paracrine activity, especially Exo biogenesis, in CSCs has been thought of as a promising therapeutic tool [231, 256]. For this purpose, specific endosomal factors such as ESCRT, sphingomyelinase, GTPase proteins, etc. can be regulated to reduce Exo biogenesis and abscission. For example, using sphingomyelinase inhibitor, GW4869, and Rab27a siRNA, Exo biogenesis and release were diminished respectively in CSCs [252, 257]. The exposure of cancer cells to dimethyl amiloride can block the acidification step inside the endosomes [258, 259]. The application of a genetic approach for the suppression or down-regulation of genes responsible for Exo biogenesis, i.e. ESCRT-III protein CHMP4B, is another anti-tumor medication [260]. The advent of nanoparticle technology can help to control Exo biogenesis, formation, and abscission. For instance, gold nanoparticles exhibit anti-Exo activity via the regulation of lipid metabolism [256]. CSC Exos can be manipulated to intensify the immune system response against tumor cells or suppress the immunosuppressive signals. Emerging evidence support the fact CSC Exos are eligible immunogenic tools for developing cancer vaccines to enhance anti-tumor immune-reactivity [261]. In this regard, the isolation of patient CSC Exos enables us to fabricate personalized vaccines for specific tumor types in the clinical setting. To select appropriate therapeutic strategies, a more profound knowledge related to CSC Exo bioactivities and challenges is imperative and warrants further research (Table 4).

**Table 4:** Challenges and overcoming of exosomes in the clinical setting

Challenging	Description	Overcoming	Ref
Heterogeneity	Different cell types and the microenvironment result in various cargo	- Developing standardized isolation and purification methods	[262–264]
Yield and purity	Risk of contamination with cellular debris or other unwanted components during isolation	Optimizing cell culture conditions and increasing the release of exosomes by: - Precise adjustment of the availability of nutrients, oxygen levels, and growth factors - Simultaneous suppression of the activity of genes that inhibit exosome biogenesis. For example, knocking down Rab4 - Optimization of downstream processing methods. including the use of specialized purification methods, such as immunoaffinity-based methods, ultracentrifugation, and size-exclusion chromatography - Bioreactors, 3D cultures, and microfluidic devices	[264–268]
Reproducibility	Ability to obtain consistent and reliable results due to inefficient separation methods, difficulties in characterization, and lack of specific biomarkers	- Establishing uniform approaches in exosome isolation and purification methods - Instrument calibration - Standardized and transparent reporting, and education	[264]
Specificity	high selectivity for isolating only exosomes while preventing the inclusion of any other extracellular vesicles or impurities	Development of methods that exclusively separate and cleanse exosomes	[264]
Large-scale manufacturing and purification	- Scalability of exosome isolation and purification methods - Complex and time-consuming process of exosome isolation - Limited production output of exosomes and high development costs - The complexity associated with scaling up the manufacturing process	- Increasing output, and standardized procedures in large-scale exosome manufacturing - To scale up the culture of anchorage-dependent cells used for exosome production, it is necessary to employ technologies that maximize available surface area - Employing well-established cell lines that release the specific exosomes alongside a rigorously defined serum-free medium allows for efficient cost utilization	[14, 269–272]
Quality control	- Need for specific biomarkers - Difficulty characterizing the exosomes	- Establishing dependable and replicable techniques for quality assurance - Standardized operating procedures combined with a streamlined closed operating system that includes a fileable quality control testing program	[5, 273]
Analysis of Complex Cargos	A significant challenge in exosome-based cancer diagnosis	- Analysis of accurate and reproducible properties of exosomes including concentration, particle size, zeta potential and exosome markers	[274, 275]
Batch-to-Batch Variation	Inconsistency in the quality and quantity of exosomes produced from different batches due to several factors, including differences in cell source or culture conditions, inefficient separation methods, and difficulties in characterization	- Standardizing the exosome production process, - Enhancing the reproducibility and reliability of clinical-grade exosomes	
Dosing	The determination of the most suitable dosage of SC-EXOs for effective cancer treatment is still being investigated. The dosing requirement could differ depending on the type of cancer, how it is infused, and the therapeutic payload contained within the exosomes	During preclinical investigations, injections containing particles ranging from 10^7^ to 10^11^ have been administrated	[276]
Route of administration	The most effective routes of administration for SC-EXOs in cancer treatment include intravenous, intraperitoneal, intra-tumoral, and subcutaneous injection	- Intravenous administration facilitates the widespread distribution of SCs-Exo throughout the body, allowing for targeted treatment of multiple tumor sites and metastases - Local injection allows for the direct delivery of SCs-Exo into precise tumor areas, optimizing their concentration at the activity site	[276]
Short Half-Life in Vivo	Constraining exosomes' therapeutic potential due to limited duration in the body with rapid recognition by: - Diverse enzymatic breakdown mechanisms in bodily fluids like blood and lymph systems break the exosome membrane - The reticuloendothelial system (RES) clears foreign particles from the bloodstream	Employing diverse strategies to bolster the stability and endurance of exosomes: - Enhancing the resilience of the exosomal membrane by coating it with protective materials or engineering it to resist enzymatic degradation - Shielding exosomes from the reticuloendothelial system by encapsulating them in biocompatible materials or nanoparticles	[197, 265]
Risk of thrombosis and hemostatic disorders	Thrombosis and homeostatic imbalance in biofluid-derived exosomes can contribute to the development of certain diseases, including cancer metastasis	Minimized the risk of thrombosis from exosome injection by: - Use of thrombolytic drugs, platelet-derived exosomes, immunoglobulin-M (IgM) antibodies, and nanomedicine	
Long-term preservation	storage conditions can impact the size distribution, quantity, contents, and cellular uptake of exosomes Ensuring the stability and functionality of exosomes over extended periods	- Lyophilizing - Employing stabilizing agents like sugars or polyethylene glycol for preservation purposes - Minimizing the freeze–thaw cycles, as repeated cycles may affect the morphology and functionality of exosomes	[197, 277]

### Clinical application of SC Exos in cancers

SC Exos possess unique features that make them suitable for therapeutic purposes in cancer treatment.

#### SC Exos as the natural delivery platform

The unwanted impact of chemotherapy protocols on non-targeted tissues and organs is a challenging issue in cancer patients [278]. To achieve anti-tumor features, it is essential to use elevated doses of drugs despite the possibility of high toxicity for non-target cells [20]. The release of chemotherapeutics using Exos has been thought of as a more efficient approach to circumvent these side effects. SC Exos are valid delivery tools with suitable interaction between the homogenous and heterogeneous cell types [279, 280]. Compared to synthetic nanoparticles such as liposomes, Exos are non-immunogenic with a specific life span [279]. Due to the dynamic distribution of Exos and different uptake systems, these nanoparticles can be used in personalized medicine. These features make possible the load of several therapeutics onto the exosomal lumen and decoration with specific ligands (integrins) to increase on-target delivery and make them cross natural barriers such as BBB [22, 281]. The existence of a lipid bilayer around the therapeutic compounds keeps them away from degradation inside the TME [281]. Besides, these features, the load of chemotherapeutics inside Exos reduces the efficient dose and thus possible side effects [282, 283]. The target molecules can be loaded onto the Exos by using several strategies. In passive cargo loading, the compounds are trapped using a diffusion process like incubation, but the loading efficiency is low [284, 285]. Compared to passive methods, in active cargo loading the compounds are actively injected into the Exo lumen using techniques such as ultrasound and electroporation. These approaches can exert reversible injury to the exosomal membrane. However, the load of the drug, retainability, and stability are high in this method compared to passive drug loading [285, 286]. In an alternative approach, the parent SC is manipulated genetically before Exo isolation or co-cultured with the target molecules, leading to the sequestration of therapeutic compounds onto the Exos in the conditioned medium [284, 285]. Of course, the application of these methods depends on the type of cargo. In the case of drug loading using electroporation or ultrasound approaches, the aggregation of proteins and genetic materials is so high that can increase the possibility of Exo membrane injury and delivery efficiency [284]. Emerging data have indicated a load of small molecules, mRNAs, and proteins with tumoricidal properties onto Exos for therapeutic purposes [202]. Further studies are mandatory to find suitable loading techniques with minimum damage to the Exo structure. The identification of valid anti-tumor cargoes with possible translation capacity to clinical settings is at the center of the debate.

#### SC Exos for targeted cancer therapy

Recently, scientific society has concentrated on finding novel and sophisticated methods for the direction of SC Exos toward anaplastic sites to yield better therapeutic outcomes [287]. Tumor cells are at the center of targeted therapy by aiming certain factors required for dynamic growth, proliferation, and survival which are not overactive in normal healthy cells [288]. Compared to conventional therapies which target all dividing cells, targeted therapy compounds specifically aim for certain effectors in tumor cells. Using engineering tools, it is possible to develop specific Exo types with higher on-target delivery approaches. For instance, tumor-targeting proteins, peptides, or antibodies can increase the delivery efficiency in tumor cells compared to normal cells [221, 289]. Despite the superiority of Exo-based approaches compared to whole-cell-based therapies, cancer therapy resistance was reported in a study after the application of bone marrow MSC Exos [290]. Under such conditions, TME was remodeled and chemoresistance capacity was induced. However, the modulatory effects of bone marrow MSC Exos on CSCs have been approved by targeting specific intracellular signaling pathways or membrane-bound factors [184, 291].

Several documents have revealed the suitable tumor-homing capacity of MSC Exos [292]. It was suggested that MSC Exos can easily cross the BTB, and respond to gradient density of chemotactic factors [293]. This property can be intensified by the decoration of specific ligands against tumor cell receptors on the Exo surface [294]. MSC exosomal integrin α4β1 can easily interact with VCAM-1 on the tumor cells, leading to the increase of Exo uptake in TME [220]. In general, SC Exos facilitates a promising tumor-targeted therapy by offering more efficient and less harmful outcomes.

#### SC Exos as diagnostic tools

**Fig. 6:**
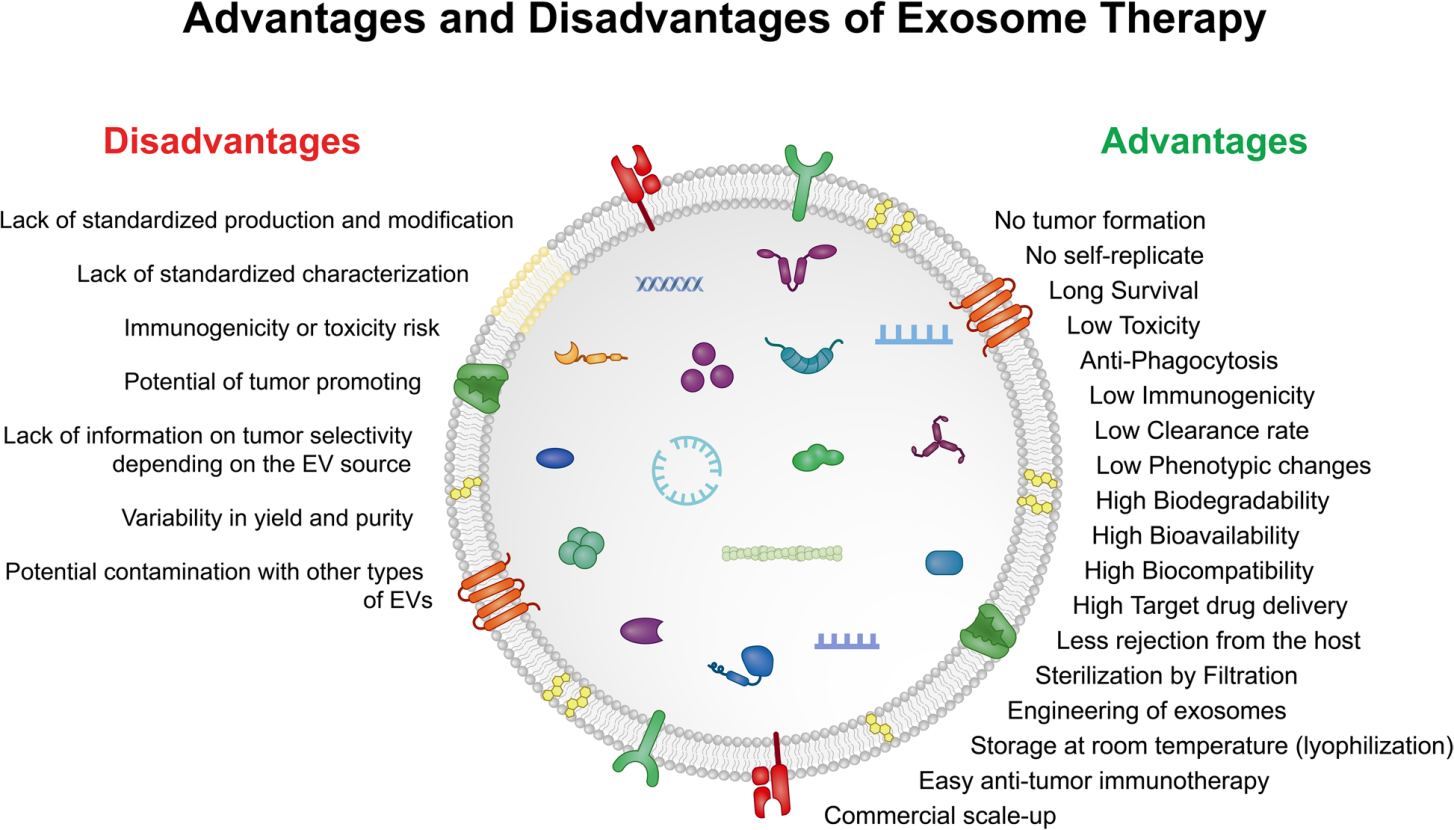
Advantages and disadvantages related to application of Exos in terms of cancers

FDA has approved several Exo-based diagnostic kits for clinical settings [295]. Like several Exo types, CSCs Exos are potential diagnostic tools. As expected, these particles can harbor specific biomolecules associated with stemness, metastasis, tumor initiation, and resistance. The real-time changes in the metabolic profile of parent cells can be precisely monitored using Exos (Fig. 6) [252]. By monitoring specific biomarkers, it is possible to predict and evaluate the efficiency of therapeutic protocols [296]. Regarding the fact that Exos can easily distribute in different biofluids they are valid non-invasive tools for the detection of anaplastic changes with suitable sensitivity and specificity. It should not be neglected that Exos are stable in ECM with heterogeneous compounds. Therefore, serial and consequential sampling enables us for precise and in-time detection of tumorigenesis [136]. Compared to Exo examination, conventional tissue sampling gives information related to a single time point and makes it difficult to make accurate decisions [296]. Despite the promising roles of circulating tumor DNA in accurate clinical detection, these molecules are released into the circulation from cells with apoptotic or necrotic changes [297, 298]. While tumor cell Exos are continuously released into the blood at all phases of tumor cell development and growth with valid data about alive cancer cells [297, 299]. Circulating tumor cells and DNAs at certain numbers and concentrations can be used as prognostic and predictive markers. Any fluctuation in these features can weaken the tumor detection rate.

As described previously, due to a lack of high-quality isolation and purification protocols, and batch-to-batch variation the bulk application of Exos has been limited in cancer patients (Fig. 6) [269]. The lack of exclusive cancer biomarkers and discrimination of cancerous and normal Exos make precise detection challengeable [269, 300]. In line with these descriptions, further investigations are mandatory for the detection of suitable Exo sources for monitoring the dynamic growth of tumor cells, and propagation. The combination of tumor cell Exos with conventional approaches can increase the sensitivity and specificity of diagnostic tools [301]. Even though, CSC Exos can reflect real genetic signatures and are unparalleled biological tools for precise cancer detection and therapy.

## Clinical trials and future perspectives

The safety and efficacy of SC Exos have been investigated for the treatment of various cancers in preclinical studies (Table 5). However, there are few clinical trials in this regard. For example, researchers at the MD Anderson Cancer Center (NCT03608631) are conducting a phase 1 study to assess the appropriate dosage and potential adverse effects of MSC Exo with Kras^G12D^ siRNA in patients with pancreatic cancer [302]. By launching another phase 1 clinical trial (NCT04592484), Codiak Biosciences aims to explore the efficacy and safety of exoSTING8, engineered Exos, in treating multiple solid tumors. Data confirmed that manipulating SC Exos through engineering approaches holds promise for future therapeutic applications [303].

**Table 5:** List of clinical trials based on Exos in cancer patients accessed on 18, January 2024

Study Title	ClinicalTrials.gov ID	Status	Study Type	Phase
KrasG12D siRNA-loaded MSC Exos (iExos) for treating patients with pancreatic cancer with KrasG12D mutation	NCT03608631	Active, not recruiting	Interventional	I
CDK-002-loaded Exos (exoSTING) in subjects with Advanced and metastatic neck squamous cell cancer, triple negative breast cancer, anaplastic thyroid carcinoma, and cutaneous squamous cell carcinoma	NCT04592484	Completed	Interventional	I and II

The future perspectives of SC Exos in cancer treatment have garnered significant interest in the scientific and medical communities. Exos can successfully combat drug resistance and ameliorate the frequently encountered side effects associated with conventional treatments [304]. Recent advancements in engineered Exo technologies provide exciting opportunities for targeted therapies by modifying surface receptors and loading specific molecules. Despite these features, more investigations are required to overcome the challenges of standardizing isolation techniques and unraveling the intricate mechanisms behind the anti-tumor effects exerted by Exos. To be specific, SC Exos are essential elements in personalized medicine strategies for cancer patients, offering improved effectiveness alongside limited toxicity. Nonetheless, we have just started along this path, and to continue, meticulously planned prospective randomized clinical trials are necessary.

## Data Availability

No datasets were generated or analysed during the current study.

## Abbreviations

ACLS4: Acyl-CoA synthetase long chain family member 4
ADAM-17: A disintegrin and metalloprotease 17
AKT: Protein kinase B
ALIX: ALG-2-interacting protein X
ANGPT2: Angiopoietin-2
ANXA6: Annexin A6
APCs: Antigen-presenting cells
BBB: Blood–brain barrier
BTB: Blood–tumor barrier
CAFs: Cancer-associated fibroblasts
CSCs: Cancer stem cells
CTDSP1: C-terminal domain small phosphatase 1
CTLA4: Cytotoxic T-lymphocyte associated protein 4
CTLs: Cytotoxic T lymphocytes
CXCR: C-X-C Motif Chemokine Receptor
DCs: Dendritic cells
E2F1: E2F transcription factor 1
ECM: Extracellular Matrix
ECs: Endothelial cells
EMT: Epithelial-mesenchymal transition
EndMT: Endothelial-mesenchymal transition
ERK: Extracellular signal-regulated kinase
ESCRT: Endosomal sorting complexes required for transport
ESCs: Embryonic stem cells
EVs: Extracellular vesicles
Exos: Exosomes
ECM: Extracellular matrix
EZH2: Enhancer of zeste homolog 2
FasL: Fas ligand
G-CSF: Granulocyte colony-stimulating factor
GJA1: Gap Junction Protein Alpha 1
gp100: Glycoprotein Gp 100
Her2/Neo (ERBB2): Erb-B2 receptor tyrosine kinase 2
HGF: Hepatocyte growth factor
HIF-1α: Hypoxia-inducible Factor 1α
HRS: Hepatocyte growth factor receptor substrate
HSP: Heat shock protein
IFN-γ: Interferon γ
ILs: Interleukins
ILVs: Intraluminal vesicles
iPSCs: Induced pluripotent stem cells
JAK: Janus kinases
JNK: C-Jun N-terminal kinases
KIT: Receptor tyrosine kinase
KLF7: Kruppel-like factor 7
LIMA1: LIM domain and actin-binding protein 1
LIMK1: LIM domain kinase 1
MAPK: Mitogen-activated protein kinase
Mart1: Melanoma-associated antigen recognized by T cells
M-CSF: Macrophage colony-stimulating factor
MDR: Multiple drug resistance;
MDSCs: Myeloid-derived suppressor cells
MEK: Mitogen-activated protein kinase kinase
MET (HGF receptor): Hepatocyte growth factor receptor
miR: MicroRNA
MMPs: Matrix metalloproteinases
MSCs: Mesenchymal stem cells
mTOR: Mammalian target of rapamycin
MVBs: Multivesicular bodies
NFE2L2: Nuclear factor erythroid 2-related factor 2 (NRF2)
NF-κB: Nuclear factor kappa-light-chain-enhancer of activated B cells
NK: Natural killer cells
NKG2D: Natural killer group 2D
nSMase 2: Neural sphingomyelinase 2 enzymes
PD-1: Programmed death-1
PDCD1: Programmed cell death protein 1
PDGFB: Platelet-derived growth factor subunit B
PGE2: Prostaglandin E2
PI3K: Phosphoinositide 3-kinases
PIAS3: E3 SUMO-protein ligase
Prkar1α: Protein kinase A regulatory subunit Iα
PTEN: Phosphatase and tensin homolog
PTK2: Protein tyrosine kinase 2
Rab: Ras-associated binding
RAF1: Rapidly Accelerated Fibrosarcoma
ROCK: Rho-associated protein kinase
RORα: RAR-related orphan receptor alpha
S100A8/A9: S100 calcium-binding proteins A8 and A9
SMAD: Suppressor of mothers against decapentaplegic
Stem cell: SCs
SCF: Stem cell factor
SDF-1α: Stromal-derived factor 1 alpha
Snail: Zinc finger protein SNAI1
SNAP23: Synaptosomal-associated protein 23
SNARE: Soluble N- ethylmaleimide- sensitive fusion attachment protein receptor
SOCS3: Suppressor of cytokine signaling 3
STAT: Signal transducers and activators of transcription
SYX-5: Syntaxin 5
TAMs: Tissue associated macrophages
TGF-β: Transforming growth factor-β
Th1: Type 1 T helper
Th17: Type 17 T helper
TIM3: T cell immunoglobulin and mucin domain-containing protein 3
TIMP2: Tissue inhibitor of metalloproteinases 2
TLR2: Toll-like receptor 2
TME: Tumor microenvironment
TNF-α: Tumor necrosis factor alpha
TRAIL: TNF-related apoptosis inducing ligand
T_reg_: Regulatory T cell
TRP: Transient receptor potential channel
TSGA10: Testis-specific gene antigen 10
TSP1: Thrombospondin 1
TXNIP: Thioredoxin-interacting protein
VAMP3/7: Vesicle-associated membrane protein 3
VEGF: Vascular Endothelial Growth Factor
VM: Vasculogenic mimicry
VPS4: Vacuolar protein sorting 4
VTA1: Vacuolar protein sorting-associated protein
VAMP: Vesicle-associated membrane protein
WNT: Wingless-related integration site
YKT6: N-ethylmaleimide-sensitive factor attachment protein receptor
Zeb: Zinc finger E-box-binding homeobox
